# Evaluation of Chronic Liver Disease: Does Ultrasound Scoring Criteria Help?

**DOI:** 10.1155/2013/326231

**Published:** 2013-09-10

**Authors:** Shaista Afzal, Imrana Masroor, Madiha Beg

**Affiliations:** Aga Khan University Hospital (AKUH), Stadium road, Karachi 74800, Pakistan

## Abstract

Noninvasive approaches for assessment of liver histology include routine laboratory tests and radiological evaluation. The purpose of our study was to determine the utility of a simplified scoring system based on routinely evaluated ultrasound features for the evaluation of chronic liver disease and correlate it with the histological findings. For this cross-sectional analytical study the data was collected prospectively by nonprobability purposive sampling technique. The ultrasound variables/parameters and their assigned scoring system that was a modified version adopted from published literature were evaluated. Sensitivity, specificity, positive and negative predictive values of the liver morphological score and combined score of liver morphology and sizes was determined using stage and grade as reference standard. Our results show a high sensitivity and PPV of liver morphological sonographic evaluation for the staging and grading of CLD respectively thus supporting it as a screening diagnostic strategy. Of the three liver morphology variables, specificity of liver surface evaluation was highest for the stage of fibrosis and grade of inflammation. The simplified ultrasound scoring system evaluated in our study is clinically relevant and reproducible for differentiating patients with CLD with mild or no fibrosis from moderate to severe fibrosis.

## 1. Introduction

The common causes of chronic liver disease are viral hepatitis, alcohol abuse, and metabolic disorders. These result in hepatocytes damage, the consequence of which may be liver fibrosis, cirrhosis, and/or hepatocellular carcinoma [1]. The disease is a substantial cause of morbidity and mortality in the developing countries. Accurate evaluation of the severity of disease is crucial for treatment planning, that is, commencement of antiviral treatment and prognostication [2].

Noninvasive approaches for assessment of liver histology include routine laboratory tests like serum markers, liver functions test, and radiological evaluation of liver. Liver histological diagnosis based on needle biopsy determines the inflammatory activity (grading), the extent of fibrosis (staging), and other comorbidities [3]. But the procedure of ultrasound guided liver biopsy is invasive with about 1% risk of significant complications like postinterventional hemorrhage, bile leak, infection, and injury to adjacent organs with less than 0.1% mortality [4]. Sampling errors may also be encountered since the liver parenchymal damage in chronic hepatitis is not homogeneous. In addition there is possibility of inter- and intraobserver variability [5]. Imaging technologies, particularly ultrasound, are inexpensive, noninvasive, readily available, and acceptable to the patient. It is routinely utilized in evaluation of spectrum of chronic liver disease as it provides useful information on the morphological alterations of the liver and organs affected as a result of portal hypertension; in addition color Doppler flow imaging provides information regarding the liver hemodynamics. The other imaging modalities like computerized tomography (CT) and magnetic resonance imaging (MRI) are also helpful, but these are expensive and require contrast administration [6]. A number of ultrasound variables based on liver morphology, hemodynamics, and different techniques of ultrasound like simultaneous use of high and low frequency transducers have been evaluated to predict the liver fibrosis stage with variable accuracy. The purpose of our study was to determine the utility of a simplified scoring system based on routinely evaluated ultrasound features for the evaluation of chronic liver disease and correlate it with the histological findings. The concept is to evolve an effective, simple to understand, applicable, and radiologically relevant scoring system for evaluation of the extent of chronic liver disease. 

## 2. Material and Methods

This cross-sectional analytical study was performed in the Department of Radiology AKUH from January 2010 to December 2011. The data was collected prospectively by nonprobability purposive sampling technique. All patients sent to the Radiology Department of Aga Khan University Hospital for ultrasound guided liver biopsy were included. Patients were excluded if the histopathology report of liver biopsy was not available and if the biopsy was performed for focal lesions or autoimmune liver disease. In addition patients unfit for liver biopsy due to jaundice, ascites, and deranged blood profiles were also excluded. Patients' confidentiality was guaranteed and maintained during the course of the study.

Prior to the biopsy real time ultrasound using Toshiba Nemio XG was performed for all patients using 3.5–5.0 MHz convex transducer by the radiologist on duty in the ultrasound interventional suit having at least 3 years of experience in performing abdominal sonography. Ultrasound of the liver was performed, both lobes of liver were evaluated, and a combined impression was derived. In addition size of liver, spleen, and portal vein was also assessed and noted. The ultrasound parameters and scoring system were explained to examining radiologist prior to the procedure and findings recorded on a standard proforma. The ultrasound variables/parameters and their assigned scoring system that was a modified version adopted from the published literature are as depicted in Table 1 [7, 8]. 

The sample for the liver biopsy was obtained from the right lobe of liver-anterior segment using Bards Tru-Cut Biopsy 18 gauge needle. The specimen was reviewed by histopathologist unaware of the ultrasound findings. 

 The histopathology reports were reviewed through the hospital information system and assessed for grading and staging of the biopsy specimen which was analyzed using the Batts and Ludwig scoring system. Grade evaluated the degree and location of inflammation and stage assessing the location and extent of fibrosis in the biopsy specimen. According to the histopathology scoring system stage 0 was described as no fibrosis and with increasing fibrosis a score of 4 was assigned for cirrhosis. For the grading score 0 described portal inflammation only and with increasing lobular inflammation and necrosis, score of 4 denoting severe diffuse hepatocellular damage with bridging necrosis [9].

For the purpose of analysis stage and grade 0 and 1 were taken as mild or no disease and stages 2, 3, and 4 as moderate to severe disease.

The ultrasound scoring system was also categorized as “A” for liver morphological evaluation comprising of liver surface, parenchymal echo texture, and edge and “B” for the combined score of liver morphology as detailed above and sizes evaluation of liver, spleen, and portal vein.

### 2.1. Sample Size Calculation

Calculated sensitivity of ultrasound for detecting chronic liver disease is 77% [10] with confidence level of 95%, margin of error 10%, and calculated sample size *N* = 115. This was done by using the following formula *N* = [*z*_1−*α*/*ε*_^2^(1 − *P*/*ε* ^2^ *P*)].

### 2.2. Plan of Analysis

Data was entered and analyzed using SPSS windows package version 19.0. Frequencies were calculated, and proportions reported for categorical variables. Mean and standard deviations calculated for quantitative variable like age. Sensitivity, specificity, positive, and negative predictive values with 95% confidence intervals were reported for ultrasound in detecting chronic liver disease in the patients taking histopathology/biopsy as gold standard. 

## 3. Results

The study population (*N* = 116) included predominantly males, 74 (64%) with a mean age of 39.54 years ± SD 12.77, range between 15 and 70 years. Data was collected of 116 patients prospectively over a period of two years from a tertiary care center, the Aga Khan University Hospital. Out of the 116 patients, 78 (67%) were hepatitis C reactive.

Sensitivity, specificity, positive and negative predicted values of the liver morphological score denoted as “A” and combined score of liver morphology and sizes denoted as “B” was determined using stage and grade as reference standard (Table 2).

Majority of patients 97 (84%) presented with normal liver size, 11 (9%) presented with an enlarged liver, and 8 (7%) presented with a liver smaller in size. The liver surface was smooth in 71 (61%), while 32 (28%) showed a mildly irregular liver surface. 13 (11%) presented with an irregular liver surface.

Liver edge was sharp in 38 (33%), mildly blunted in 66 (57%), and rest 12 (10%) showed a blunted liver edge. Portal vein was normal in 112 (97%) and dilated in the remaining of the total sample.

Sensitivity, specificity, positive and negative predicted values and *P*-value using chi-square test of the each ultrasound variable were determined using stage and grade as reference standard. {Tables 3(a) and 3(b)}.

## 4. Discussion

Chronic liver disease is a spectrum of disease manifestation leading to cirrhosis. Current development and improvement in the treatment and management options have stressed a need for prompt diagnosis of CLD to identify asymptomatic patients in a population that is high risk, for example, due to high prevalence of viral hepatitis, and hence provide a better patient outcome. Accurate estimation of the degree of hepatic damage in fibrosis or cirrhosis before the compensation becomes clinically evident is crucial for treatment, prognosis, and surveillance. The noninvasive methods to assess features of CLD include serologic fibrosis markers like fibro test, aspartate aminotransferase-to-platelet ratio index (APRI), and radiologic imaging [11]. These tests are regarded to be perfect and ideal only if these are simple, accessible cheap, and exhibit high accuracy. 

In the present study we attempted to develop a simplified scoring system based on ultrasound parameters routinely evaluated in sonographic studies and likely to be affected during the course of CLD like liver morphological appearance and the dimension of liver, portal vein, and spleen. The US scores were compared with the histopathological results of the biopsy specimen. A number of studies have utilized the ultrasound examination for the diagnosis and staging of chronic liver disease making use of different techniques like the conventional gray scale and Doppler [12, 13] to sophisticate technique of transient elastography and using contrast agent [11, 14]. Gaiani et al. [13] investigated patient with chronic liver disease for the presence of compensated cirrhosis using ultrasound scoring system and achieved the sensitivity and specificity of 78.7% and 80.2%, respectively. A comparison of prior studies using ultrasound scores for the evaluation of chronic liver disease is shown in Table 4.

Our results show a high sensitivity and PPV of liver morphological sonographic evaluation for the staging and grading of CLD, respectively, thus supporting it as a screening diagnostic strategy. The two groups of liver fibrosis that is mild/no fibrosis and moderate/severe fibrosis/cirrhosis could be differentiated using this scoring system with high sensitivity and PPV. This is likely to be related to the fact that the simplified scoring system in the present study evaluated the findings on a 3 level scale, that is, 0, 1, and 2 as compared to other studies [15] where findings were evaluated on a 4-point scale ranging from 0 to 4.

Of the three liver morphology variables, liver surface evaluation depicted specificity of 86.3% for the stage of fibrosis and 91.1% for the grade of inflammation. The result is in keeping with other studies that showed a high specificity of surface nodularity [16]. In this prospective study liver edge was also found to have a high sensitivity and specificity for detection of liver fibrosis and grades of inflammation and differed from other studies in which liver edge was not found to be specific for liver fibrosis evaluation [7]. 

In this study the cut-off value of the ultrasound score was 2 for liver morphology (category A) and 3 for combination of morphology and sizes (category B). The liver morphology score using 3 variables provided a sensitivity of 90.3%, but a sensitivity of 44.4% was achieved when all 6 variables were assessed and is lower than that reported by using 4 variables [8]. The patients with clinically decompensated CLD were excluded in the present study to maximize the efficacy of the ultrasound examination. But in addition to the US signs for assessing liver parenchyma, signs consistent with advanced liver disease like enlarged spleen, shrunken liver, and portal hypertension were also evaluated for their presence in nonsymptomatic patients. The number of patients diagnosed as stage IV fibrosis on histopathology in the present study is 19 (16.4%), while on sonography the frequency of small shrunken liver and splenomegaly is 8 (7%) and 10 (8.6%), respectively.

This study has a few limitations. The study results show high sensitivity, but the specificity is low, and hence there is a need to come up with further research to get better diagnostic accuracy. This can be achieved by addressing factors such as intra and inter observer variability, quality assurance of the technique and equipment of ultrasound. Since liver histology was taken as gold standard in this study, the possibility of sampling errors and inter- and intraobserver variability in assessment of biopsy specimen cannot be ruled out and may have also affected our results. 

Presence of hepatic steatosis significantly affects the liver parenchymal appearance, but this finding was not assessed in the US evaluation of the study group.

## 5. Conclusion

The simplified ultrasound scoring system evaluated in our study is clinically relevant and reproducible for differentiating patients with CLD with mild or no fibrosis to moderate to severe fibrosis. Since we also evaluated the sensitivity of these parameters for grading, it is also helpful in determining the prognosis and best possible therapeutic option. 

## Figures and Tables

**Table 1:** Ultrasound variable and scores.

Variables	Score 0	Score 1	Score 2
Liver parenchymal echotexture	Homogenous/fine	Coarse	Highly nonhomogenous/coarse
Liver surface	Smooth	Irregular	Nodular
Liver edge (inferior margin right lobe)	Sharp (acute)	Blunted	Rounded
Liver size	Normal	Enlarged (>15 cm mid clavicular line)	Shrunken (<10 cm in mid clavicular line)
Portal vein diameter	Normal	Dilated (>13 mm)	
Spleen size	Normal	Enlarged (>13 cm)	

**Table 2:** Accuracy of US scoring system.

	Histopathology	Sensitivity	Specificity	PPV	NPV
A = liver morphological score	Stage	90.3%	47.7%	73.9%	75%
	Grade	84.1%	44.1%	78.4%	53.6%
B = liver morphological score + liver, spleen, and portal vein size	Stage	44.4%	88.6%	86.5%	49.4%
	Grade	41.5%	91.2%	92%	39.2%

**(a):** 

Variable	Sensitivity (95% CI∗)	Specificity (95% CI∗)	^∧^PPV (95% CI∗)	^§^NPV (95% CI∗)	^¤^ *P* value
Liver edge	84.72 (0.74, 0.92)	61.36 (0.46, 0.75)	78.21 (0.67, 0.86)	71.05 (0.55, 0.84)	0.000
Liver surface	54.16 (0.42, 0.65)	86.36 (0.72, 0.94)	86.66 (0.72, 0.94)	53.52 (0.41, 0.65)	0.000
Liver texture	75 (0.63, 0.84)	66 (0.49, 0.79)	78.26 (0.66, 0.86)	61.7 (0.46, 0.75)	0.000
Liver size	12.5 (0.06, 0.22)	77.27 (0.61, 0.88)	47.3 (0.25, 0.70)	35.05 (0.26, 0.45)	0.149
Spleen size	9.72 (0.04, 0.19)	93.2 (0.80, 0.98)	70 (0.35, 0.91)	38.68 (0.29, 0.48)	0.589
Portal vein diameter	5.55 (0.01, 0.12)	100^(*∞*)^	100^(*∞*)^	39.28 (0.30, 0.48)	0.112

^∧^PPV: positive predictive value.

^§^NPV: negative predictive value.

(95% CI∗): 95% confidence internal.

^¤^ *P* value: chi-square test *P* value.

^(*∞*)^: Cannot be calculated because either of one cell contains a “zero."

**(b):** 

Variable	Sensitivity (95% CI∗)	Specificity (95% CI∗)	^∧^PPV (95% CI∗)	^§^NPV (95% CI∗)	^¤^ *P* value
liver edge	80.5 (0.70, 0.88)	64.71 (0.46, 0.79)	84.62 (0.74, 0.91)	57.89 (0.41, 0.73)	0.000
liver surface	51.22 (0.40, 0.62)	91.18 (0.75, 0.97)	93.33 (0.81, 0.98)	43.66 (0.32, 0.55)	0.000
liver texture	72 (0.61, 0.81)	70.6 (0.52, 0.84)	85.51 (0.74, 0.92)	51.06 (0.36, 0.65)	0.000
liver size	14.63 (0.08, 0.24)	79.41 (0.61, 0.90)	63.16 (0.38, 0.82)	27.83 (0.19, 0.38)	0.430
spleen size	9.76 (0.04, 0.18)	94.12 (0.78, 0.98)	80 (0.44, 0.96)	30.28 (0.22, 0.39)	0.499
portal vein diameter	4.88 (0.01, 0.12)	100^(*∞*)^	100^(*∞*)^	30.36 (0.22, 0.39)	0.190

^∧^PPV: positive predictive value.

^§^NPV: negative predictive value.

(95% CI∗): 95% confidence internal.

^¤^ *P* value: chi-square test *P* value.

^(*∞*)^: Cannot be calculated because either of one cell contains a “zero."

**Table 4:** Comparison with prior studies of validity of ultrasound score in the evaluation of chronic liver disease.

Study	Number of patients	Characteristics	Sensitivity	Specificity
Gaiani et al. [13] 1997	212	US scoring comprising of seven morphological and hemodynamic hepatic parameters	82%	79%
Hung et al. [8] 2003	210	US scoring comprising of liver surface, parenchyma, vascular structure, and spleen size	HBV-related cirrhosis 77.8% HCV-related cirrhosis 82.4%	HBV-related cirrhosis 92.5% HCV-related cirrhosis 70.7%
Choong et al. [15] 2012	156	Three hepatic features assessed	53% (combined score liver surface and edge)	94% (combined score of surface and texture)
Afzal et al. 2013	116	US scoring system based on six variables	Liver morphology 90.3% (stage) 84.1% (grade) Liver morphology + size of liver, spleen, and PV 44.4% (stage) 41.5% (grade)	Liver morphology 47.7% (stage) 44.1% (grade) Liver morphology + size of liver, spleen, and PV 88.6% (stage) 91.2% (grade)
